# Editorial

**DOI:** 10.34763/jmotherandchild.20212501.edit.2021_25_01

**Published:** 2021-10-11

**Authors:** Aleksandra Jezela-Stanek

**Affiliations:** 1 Department of Genetics and Clinical Immunology National Institute of Tuberculosis and Lung Diseases Warsaw, Poland

## Editorial

The editors of the *Journal of Mother and Child/Medycyna Wieku Rozwojowego* invite you to read the latest issue of our quarterly, the first in 2021. The topics discussed in this issue encompass health problems in preterm-born children and the effect of continuous supportive telephone counselling on improving breastfeeding self-efficacy; adverse birth outcomes in Ethiopia; and health complaints among Polish adolescents. Other topics include a clinical report of G6PD deficiency with Harilaou variant and an interesting original paper presenting a molecular analysis of large or giant congenital melanocytic nevi. Overall, we offer seven original articles.

This issue’s first article discusses a study carried out in a group of 43 children prematurely born from singleton, twin, and triplet pregnancies and assesses their functional development between the ages of 2 and 2.5 years. Interestingly, no relationship was found between the multiplicity of pregnancies and the functional development of premature babies born between the 32nd and 36th weeks of gestation. Contrarily, residence, lack of family support during childbearing, pregnancy type, short interpregnancy interval, current obstetric complications, and a number of antenatal care (ANC) visits were identified as determinants of adverse birth outcomes in Ethiopia. The authors of that study recommended improving family support, lengthening interpregnancy intervals through family planning counselling and provision, and ensuring the recommended number of ANC visits.

Two other clinically significant papers on prematurity are also presented. One investigates the effect of continuous, supportive telephone counselling on improving breastfeeding self-efficacy in mothers with late preterm infants is one of these. In the second paper, Asfour and colleagues give a meaningful description of a previously unrecognized adverse drug effect associated with daptomycin administration. Monitor of alanine aminotransaminase and prothrombin time seem to require attention.

Medication is the topic of another article as well. Over-the-counter (OTC) drugs are becoming increasingly popular, leading the authors to study the habits and knowledge of parents regarding the use of OTC antipyretics for their offspring and consider the demographic and socioeconomic characteristics of the families. The presented data seem quite worrying; thus Pyznar et al. suggest educating caregivers and building parents’ awareness that they should take an active role in their child’s treatment. According to the authors, “It would be useful to create generally available recommendations for home treatment.”

We continue with another excellent original paper dedicated to adolescents’ health, describing the trend of subjective health complaints of Polish adolescents compared to peers in 30 other countries and ranking all countries on a new measure. The authors suggest the HBSC-SCL index as useful for monitoring change in adolescent mental health. The proposed method of ranking may allow a broader view of the differences and similarities between countries and help to identify those performing unfavourably against cross-country patterns.

“Urban Health and Nutrition Day or Only Immunization Day?” is a must-read for anyone interested in the mission of Urban Health and Nutrition Day. One can learn the barriers and bottlenecks, as perceived by the community health workers providing this service in the selected city of Nagpur, Maharashtra, India.

This issue also includes the first molecular analysis of large/giant congenital melanocytic nevi (CMN) in Polish patients, where the authors support the hypothesis of the *NRAS* variant in codon 61 importance as frequent, recurrent genetic causes of large/ giant CMN. Finally, we recommend an interesting and detailed article not to be missed by any researcher involved in inherited enzymopathies: a new case of G6PD Harilaou variant in a Greek male neonate, who suffered severe intrauterine haemolysis and passed away 39 hours after birth.

We hope that the presented papers meet your expectations and encourage you to read future issues of our journal.

Aleksandra Jezela-Stanek MD, PhD, Asst Prof Department of Genetics and Clinical Immunology National Institute of Tuberculosis and Lung Diseases Warsaw, Poland

